# Gendered anthropomorphism in human–robot interaction: the role of robot gender in human motivation in task contexts

**DOI:** 10.3389/fpsyg.2025.1593536

**Published:** 2025-06-25

**Authors:** Yi Zhu, Ling Su, Lijing Zheng

**Affiliations:** ^1^School of Management, Guangzhou College of Technology and Business, Guangzhou, China; ^2^Institute for New Quality Productive Forces and GBA, Guangzhou College of Technology and Business, Guangzhou, China; ^3^Business School, Shanghai Jian Qiao University, Shanghai, China

**Keywords:** HRI, anthropomorphism, group process, AI, human–AI interaction (HAII)

## Abstract

This study investigates how gendered anthropomorphism in robots influences human motivation to undertake challenging tasks within human–robot collaborative settings. Through two experiments—a survey-based experiment (Study 1, *N* = 169) and a behavioral experiment (Study 2, *N* = 130), we observed how a collocated female- versus male-gendered robot assistant affects participants’ willingness to accept a challenging task. Results revealed that interactions with female-gendered robots elicited significantly greater willingness to undertake a challenging task compared to male-gendered counterparts. This finding advances our understanding of human motivation in modern workplace environments that integrate robotic technologies, and underscores the critical role of gender cues in robot design, particularly in collaborative settings where task engagement and performance are prioritized.

## Introduction

The rapid advancement of artificial intelligence (AI) has fundamentally influenced people’s daily life. The field has witnessed an unprecedented surge in the development of AI technologies, manifesting in application domains such as intelligent cognitive architectures (e.g., DeepSeek-V3, GPT-4o) and domestic service robots (e.g., RoboRock S8 Pro Ultra). Notably, many AI-embedded products demonstrate humanlike characteristics in terms of appearance, motivation, and emotional sensing capabilities (Broadbent, 2017; Huang and Rust, 2022; Wan and Chen, 2021).

In academia, human-robot interaction (HRI) research systematically examines how anthropomorphism influences user experience and emotional responses (Fink, 2012; Huang and Rust, 2022; van Straten et al., 2020; Wan and Chen, 2021; Złotowski et al., 2015), and how social skills, mood, and wellbeing may benefit from social-robot-based interventions (Duradoni et al., 2021). Central to these inquiries is the observation that people tend to imbue robots with humanlike characteristics (e.g., motivation, emotion), and tend to interact with robots in ways that resemble human-to-human relationships. For instance, people have the tendency to extend real-life psychological dynamics toward artificial entities (Russo et al., 2021), robots designed with culturally coded feminine or masculine features (e.g., vocal pitch, body shape, or names) elicit stereotypical expectations that influence compliance, trust, and role allocation (Eyssel and Hegel, 2012; Nass and Brave, 2005). This phenomenon is particularly salient in HRI, where anthropomorphic cues can shape trust, engagement, and compliance, thereby mediating collaborative task performance (Kim and McGill, 2011).

The present investigation addresses a critical gap in HRI literature by examining the following research question: How does a robot’s gender influence human motivation to undertake challenging tasks? This inquiry holds particular relevance in achievement contexts where task complexity often exceeds individual cognitive capacity. We propose that anthropomorphic design elements, especially gender cues, may potentially shape human motivation to engage with challenging objectives, as it increases the likelihood of successfully completing such tasks. Focusing on appearance-based anthropomorphism, the present research examines how gendered robot appearance anthropomorphism influences people’s motivation to undertake challenging tasks.

### Robot appearance anthropomorphism

People often anthropomorphize intelligent machines, forming partnership with them that closely resemble human-human team interactions (Burgoon et al., 2016; Nass et al., 1995; Nass et al., 1994). In recent years, HRI research emphasizes anthropomorphic design, aiming at enabling robots to operate into human social environments such as healthcare, education, and domestic assistance (Bartneck et al., 2009; Broadbent et al., 2013).

Anthropomorphism is defined as the attribution of human characteristics, intentions, or emotional states to non-human entities (Epley et al., 2007; Fink, 2012; Wan and Chen, 2021). It facilitates people’s engagement in human-technology interactions during complex tasks (Sheehan et al., 2020), similar to their interactions with human counterparts. Humanoid robots, such as Unitree’s H1, SoftBank’s Pepper, and Boston Dynamics’ Atlas, exemplify anthropomorphic designs that enable a diverse range of functions by mirroring human characteristics and capabilities (Walliser et al., 2017).

However, the relationship between robots’ anthropomorphic appearance and user acceptance follows a curvilinear pattern. While moderate human-likeness enhances user acceptance, excessive morphological realism triggers the *uncanny valley effect*, a well-documented phenomenon where near-human replicas provoke visceral aversion (MacDorman and Chattopadhyay, 2016; Mathur and Reichling, 2016). For instance, MacDorman and Ishiguro (2006) revealed that photorealistic robot faces elicit stronger discomfort than more machine-like counterparts. Thus, it is of significance to optimize anthropomorphic design aiming to mitigate uncanny valley risks while preserving the social affordances of human-like design (Kim et al., 2019; Mathur et al., 2020).

### Robot gendered anthropomorphism

Gender stereotypes traditionally characterize women as warmer and men as more competent, a pattern well-documented in social psychology research (Eagly and Karau, 2002; Eagly and Wood, 1999; Fiske et al., 2002). These biases extend to HRI, where people ascribe gender stereotypes to robots through cues such as appearance, voice, and naming (Borau, 2024; Perugia and Lisy, 2023). For instance, Eyssel and Hegel (2012) demonstrated that robots with female traits are perceived as warmer but less competent, while male-appearance robots are viewed as more authoritative—a direct reflection of human gender norms.

Such gender stereotypes may shape perceptions of robots’ suitability for specific tasks. Studies show that male-gendered robots are often favored in technical, leadership, or authoritative contexts, whereas female-gendered robots are preferred in caregiving or service contexts (Carpenter et al., 2009; Sandygulova and O’Hare, 2018). While aligning robots with familiar social scripts may enhance user engagement, this practice risks entrenching harmful stereotypes and reducing acceptance when robots deviate from gendered expectations (Nomura et al., 2006). For instance, anthropomorphic design of the front of cars—such as headlights resembling “eyes” or grilles resembling “mouths”—can trigger gendered anthropomorphism, with users attributing traits like a “friendly female” or “aggressive male” character to inanimate objects. These perceptions, in turn, influence human behavior, including driving habits or brand loyalty (Waytz et al., 2014). Such findings underscore the dual-edged role of gendered anthropomorphism in HRI, necessitating ethical considerations in design to balance relatability with equity.

### Gendered anthropomorphism and human motivation to undertake challenging tasks

Specific and challenging tasks/goals can direct attention, mobilize effort, and foster persistence, leading to higher performance (Locke and Latham, 1990; Locke and Latham, 2006). Thus, mobilizing employees to accept and accomplish challenging tasks is critical for personal development and organizational success. In modern workplaces incorporating humanoid robots, understanding how robotic anthropomorphism influences employee’s motivation to accept challenging tasks becomes critically important.

Research suggests that motivation to undertake challenging tasks is influenced by the degree of anthropomorphic appearance in robots. In a recent study, Wang et al. (2025) demonstrated that robots with moderate anthropomorphism elicited greater willingness to accept a challenging task compared to hyper-realistic counterpart, a finding consistent with the “uncanny valley” effect documented in literature (Kim et al., 2019; Mathur et al., 2020; Mathur and Reichling, 2016). While excessive human-likeness often triggers negative affective responses (Ferrari et al., 2016; Ho and MacDorman, 2010; Müller et al., 2021; Pelau and Ene, 2018; Yogeeswaran et al., 2016; Yu, 2020), moderate anthropomorphism enhances task motivation during cognitively demanding tasks (Kim et al., 2022).

Furthermore, gendered anthropomorphism may also influence people’s motivation to undertake challenging tasks. Although gender cues of robots may result in gender-competence stereotyping beliefs as mentioned above (Duan et al., 2024; Loideain and Adams, 2020; Otterbacher and Talias, 2017; Perugia and Lisy, 2023), such stereotype may not necessarily translate to HRI in task-oriented contexts. AI-powered virtual personal assistants like Alexa, Cortana, and Siri are overwhelmingly designed with female-gendered names, voices, and personas (Loideain and Adams, 2020). Similarly, physical service robots already deployed in fields like hospitality or caregiving are often anthropomorphized as female in appearance (Alesich and Rigby, 2017). This feminization of assistive technologies may reinforce perceptions of robots as inherently suited to domestic or service roles. Such design choices could paradoxically subvert gender-competence stereotypes in task-oriented contexts, where users might prioritize functional performance over gender-competence stereotype when evaluating robotic capabilities. For instance, Carpinella et al. (2017) found that female robots were ascribed higher competence ratings and higher warmth ratings than male robots. Kapteijns and Graaf (2024) observed that female-voiced robots were ascribed higher competence than male-voiced robots.

### The present study

The present study combined a survey-based experiment (Study 1) and a behavioral experiment (Study 2) to investigate how robot gendering influences challenging task acceptance in human-robot collaboration. Survey-based experiment is a methodology widely validated in social and behavioral research (Aguinis and Bradley, 2014; Atzmüller and Steiner, 2010; Evans et al., 2015). This approach is particularly suited to HRI investigations, due to its capacity to systematically manipulate independent variables while retaining rigorous control over experimental conditions. Study 1 was part of a large project (Wang et al., 2025) in which participants were presented with a scenario depicting a human-robot team is faced with the choice of whether to accept a challenging task in exchange for an intriguing reward. After reading the scenario, participants were asked to imagine themselves collaborating with a humanoid robot partner, shown as either male or female. In the behavioral experiment, participants decided whether to undertake a cognitively demanding computer-based task with the assistance of a robot partner, again depicted as male or female. In both experiments, the robot assumed an assistive role.

Existing literature suggests that female voices and personas are preferred in assistive roles (Nass and Brave, 2005) and that female robots are rated higher in warmth and task collaboration (Carpenter et al., 2009; Siegel et al., 2009). Given these findings, it is reasonable to argue that female robots are perceived as more trustworthy than male robots when performing assistive functions. Furthermore, research indicates that interpersonal trust enhances employee task motivation (Costa et al., 2018; Edmondson, 1999). Building on these foundations, we hypothesized that participants in the female robot partner condition would demonstrate greater willingness to accept a challenging task compared to those in the male robot condition.

## Study 1

### Methods

#### Participants

A total of 169 native Chinese students from a public university in southern China were recruited and randomly assigned to either the female robot condition (*N* = 94, 46 male, *M*_age_ = 18.20, *SD*_age_ = 1.19) or the male robot condition (*N* = 75, 41 male, *M*_age_ = 18.24, *SD*_age_ = 0.66). These students were recruited from two classrooms, each representing a different experimental condition. In the absence of a precise effect size estimate, we determined our sample size based on previous research (Kim et al., 2019), which indicated that 53 participants per condition would be sufficient. However, the final sample size was ultimately constrained by the number of available participants attending the classes. Moreover, to ensure the validity of the experiment, all participants were undergraduate students whose majors were unrelated to psychology or robotics. Notably, all participants were university students, which limited the sample’s representativeness. All participants volunteered to participate in the experiment and received 5RMB (1USD = 7.2RMB) for participation.

#### Materials

**Figure 1:**
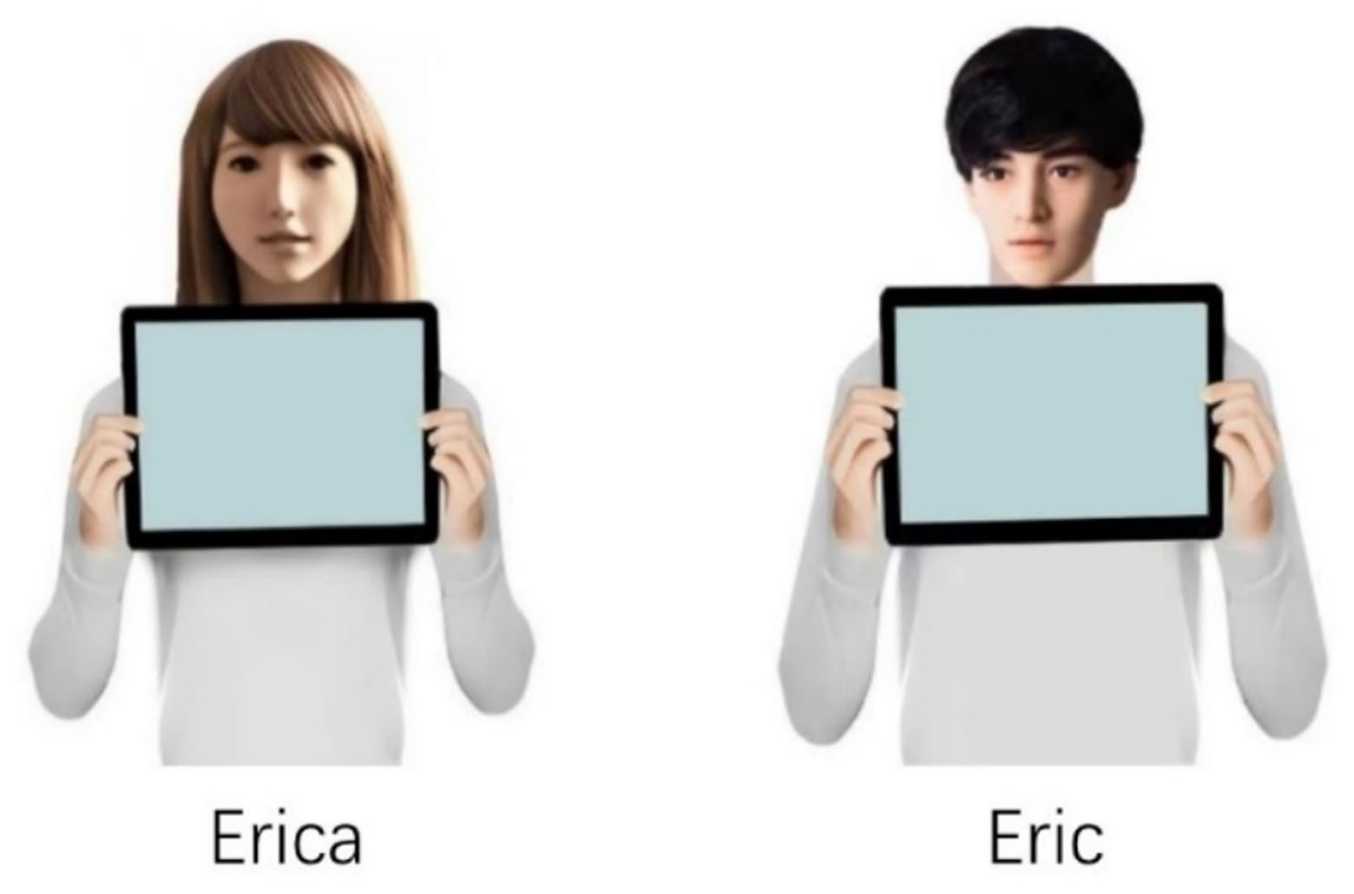
Images of robots used in the present study.

Participants were presented with a scenario in which they imagined themselves as a technician at KC, an IT company, faced with a decision to accept a challenging task in exchange for an attractive reward. Participants were informed that they would collaborate with a robot partner (named Erica or Eric, depicted in Figure 1) applied to assist in the task. The robot’s capabilities depicted in the scenario were modeled after a real-world AI tool used by an IT company to automate the review of loan application data. Participants were instructed to envision working with this robot to complete the task, leveraging its analytical skills while managing their own responsibilities. The scenario is introduced as follows:

KC is an IT company whose core business is to help banks conduct on-site inspection of information provided by companies applying for loans. KC follows the requirements of banks to collect and review information provided by those companies (information that can prove the real existence of the enterprise, such as the building name plate of those companies). Recently, KC temporarily received a business from a bank to help check information provided by their clients. A task that normally takes months must now be completed in just one—making it urgent and extremely difficult. Technician will complete the task with the help of a robot partner (see the picture below). Technician can decide whether to accept the task, and they can opt out of the task, and it will not affect their performance. Accomplishment of the task will bring additional bonuses.

The female robot, Erica, was sourced from the ABOT database^1^, a resource developed by researchers at Brown University. This database catalogs over 250 real-world anthropomorphic robots designed for research or commercial use, each rated on a 0–100 anthropomorphism scale across three dimensions: body-manipulators, face, and surface. Erica scored 89.6, indicating the high anthropomorphism. To ensure parity in anthropomorphism, the male robot, Eric, was designed based on the three dimensions, closely matching Erica’s human-likeness. In a manipulation check (Wang et al., 2025), adapted from Kim and McGill (2011), participants rated Erica (*M* = 5.20) and Eric (*M* = 5.13) similarly on items assessing anthropomorphism (e.g., “The robot looks like a person” and “The robot looks alive”). Both scored significantly higher than Pepper, a robot with moderate anthropomorphic features, which confirms the suitability of the images used in the present study.

Finally, motivation to undertake challenging tasks operationalized as participants’ willingness to accept a challenging task, assessed using a two-item measure: “I would love to accept this task” and “I’m very interested in taking this task,” *r* = 0.77, *p* < 0.001. Responses to this scale was given on a 9-point scale ranging from 1 (*completely disagree*) to 9 (*completely agree*).

### Results

**Figure 2:**
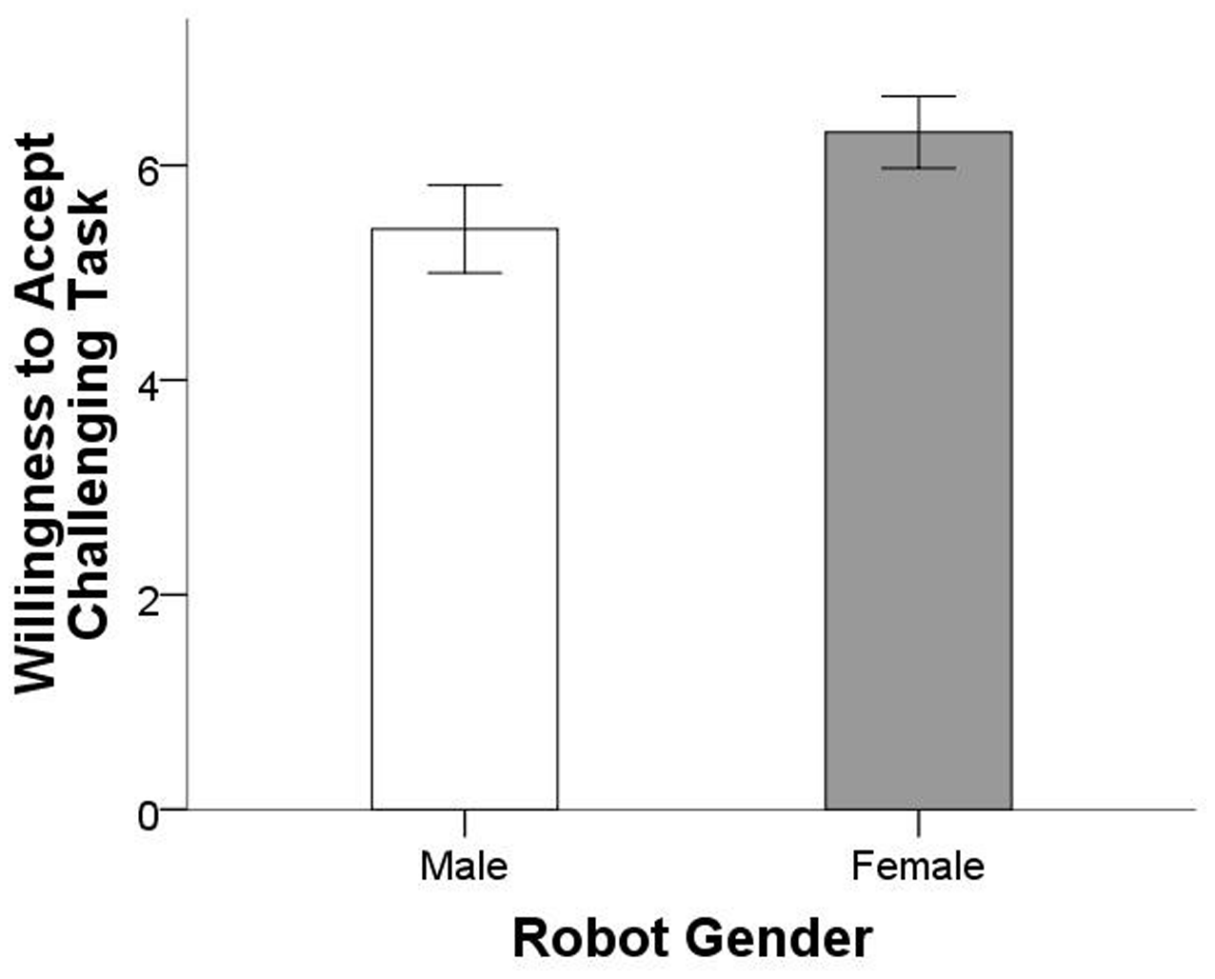
Mean willingness to accept challenging task for the two conditions. Error bars show 95% confidence intervals.

Figure 2 displays the mean willingness to accept the challenging task for the two conditions. Result showed that participants in female robot condition (*M* = 6.309, *SD* = 1.635) showed significantly higher willingness to accept challenging task (*M* = 5.407, *SD* = 1.774), *F* = 11.770, *p* = 0.001 (two-tailed), η_p_^2^ = 0.066, thus confirming our hypothesis that participants in the female robot partner condition were more willing to accept the challenging task compared to those in the male robot condition.

## Study 2

In Study 1, we demonstrated that participants interacting with a female robot exhibited greater willingness to accept a challenging task compared to those interacting with a male robot. Building on this result, Study 2 sought to replicate this finding using a behavioral measure within an experimental setting. This behavioral experiment required active engagement with cognitively demanding tasks. This paradigm offers enhanced ecological validity by capturing participants’ behavioral response rather than hypothetical scenario responses.

### Methods

#### Participants

A total of 130 students from a public university in southern China were recruited and randomly assigned to either the female (*N* = 75, 41 male, *M*_age_ = 21.76, *SD*_age_ = 0.87) or the male (N = 85, 35 male, *M*_age_ = 21.82, *SD*_age_ = 0.77) robot conditions. These students were recruited from two classrooms, each representing a different experimental condition. We determined the sample size for this study using the same method as in Study 1. All participants were undergraduate students whose majors were unrelated to psychology or robotics. All participants volunteered to participate in the experiment and received 20RMB (1USD = 7.2RMB) for their participation.

#### Experimental procedure

This study was conducted in a controlled computer lab. Upon arrival, participants were assigned predetermined computer stations. The experimenter provided an overview of the task: a cognitive task involving 20 graphical reasoning questions, each with a single correct answer. Participants were instructed to work with a robot assistant that would offer answer prompts throughout the task. Emphasis was placed on responding as quickly and accurately as possible. The session included a practice phase (5 questions) followed by a formal phase (20 questions).

**Figure 3:**
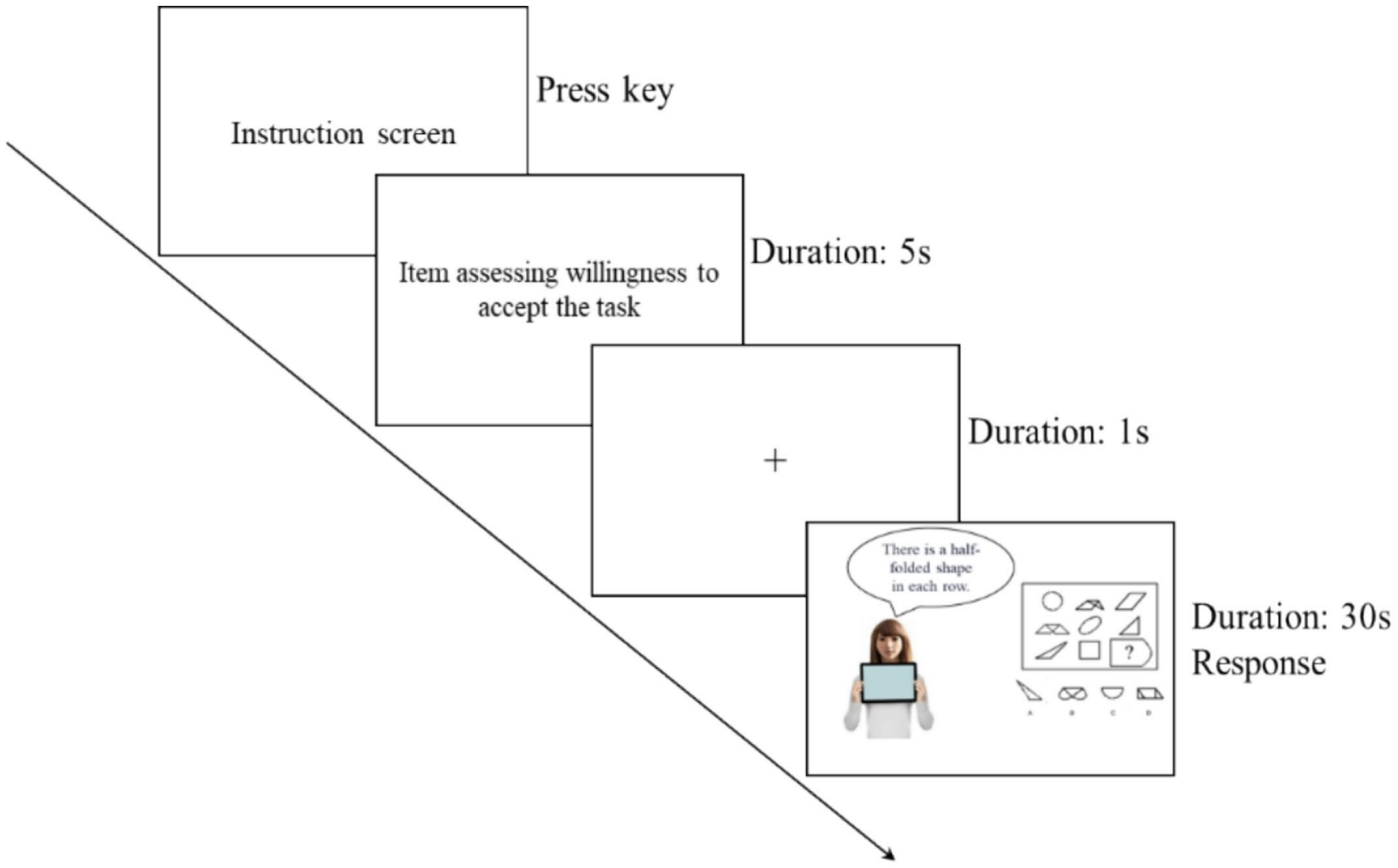
The cognitive task.

Participants were also shown via their computer screen the detailed of the task, including a practice phase, formal phase, the robot assistant they would collaborate with, as well as the task (Figure 3). After carefully reading the instruction screen, they were instructed to press the spacebar to the next screen in which they completed an item assessing their willingness to accept the task on a 9-point scale anchored at 1 (“very unwilling to perform the task”) and 9 (“very willing to perform the task”). Then participants proceeded to the task. Each trial began with a 1-s fixation cross (“+”) followed by the robot partner and the target question. Participants had 30 s per question to submit answers (via ABCD keys); unanswered items were skipped and excluded from accuracy calculations.

Before beginning the formal task, participants completed a practice session to familiarize themselves with the interface and interaction with the robot. The robot was positioned on the left screen, while the question appeared on the right. Participants could repeat the practice phase as needed; they advanced to the formal phase once they confirmed understanding of the experimental procedure. Participants were informed that the top 10% of participants with the best task performance will receive additional rewards.

After the cognitive task, participants complete a set of questionnaires measuring their robot trust, robot capability, robot attractiveness.

#### Materials

The experiment utilized the same robot images (i.e., Erica, Eric) as Study 1 (Figure 1). We administered a challenging task comprising of 20 Graphic Reasoning Questions, a task format commonly featured in standardized exams such as China’s Civil Service Exams. These questions evaluate candidates’ logical reasoning, analytical thinking, and spatial–visual processing skills. For the selection process, twenty participants evaluated the perceived difficulty of 40 candidate questions. The formal experimental task incorporated the 20 most challenging items that met dual criteria: ranking in the top 50% for difficulty ratings and requiring completion times exceeding 60 s. This task was supplemented with 5 practice questions for task familiarization. This task was designed using E-Prime 3.0.

Inspired by previous research (Lee and Liang, 2016), the ostensibly intelligent robot helped the participants by providing text-based suggestions to solve a series of cognitive tasks (Figure 3). This design is to simulate real-world human-robot collaboration scenarios, specifically examining how gendered anthropomorphism influence willingness to accept challenging task.

#### Measures

After the cognitive task, participants complete questionnaires measuring their robot trust, robot capability, and robot attractiveness. Robot trust was assessed with two items (“The robot assistant is dependable” and “The robot assistant is trustworthy”), *r* = 0.84, *p* < 0.001. Robot capability was assessed by three items (“The robot assistant is smart,” “The robot assistant is knowledgeable,” “The robot assistant is capable”), *α* = 0.948. Robot appearance attractiveness was assessed with two items (“The appearance of robot assistant is attractiveness,” “The robot assistant is likeable”), *r* = 0.610, *p* < 0.001. Responses to these scales were given on a 9-point scale ranging from 1 (*completely disagree*) to 9 (*completely agree*).

### Results

**Figure 4:**
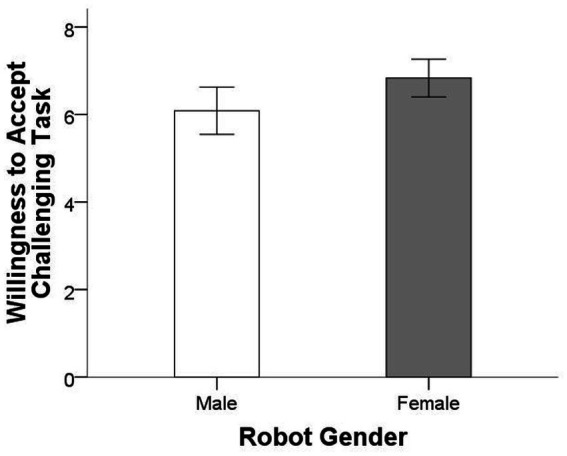
Mean willingness to accept challenging task for the two conditions. Error bars show 95% confidence intervals.

Figure 4 displays the mean willingness to accept the challenging task for the two conditions. Result again revealed a statistically significant difference in willingness to accept challenging tasks between the female (*M* = 6.83, *SD* = 1.669) and male (*M* = 6.09, *SD* = 2.263) robot conditions, *F* = 4.464, *p* = 0.037 (two-tailed), η_p_^2^ = 0.034 (Figure 4). Although the effect was less robust than in Study 1, the finding further supported hypothesis. Moreover, results also showed a significant difference in terms of robot trust between the female (*M* = 6.767, *SD* = 1.531) and male (*M* = 5.971, *SD* = 1.539) robot conditions, *F* = 8.669, *p* = 0.004 (two-tailed), η_p_^2^ = 0.063, suggesting that higher willingness to accept challenging task in the female robot condition could be driven by trustworthiness of robot. Moreover, robot capability (*M*_female_ = 6.200 vs. *M*_male_ = 6.024, *F* = 0.296, *p* = 0.588 (two-tailed), η_p_^2^ = 0.002) and robot appearance attractiveness (*M*_female_ = 5.600 vs. *M*_male_ = 5.557, *F* = 0.022, *p* = 0.883 (two-tailed), η_p_^2^ = 0.000) between the two conditions were not significantly different.

We then examined whether robot trust mediated the effects of robot gender on willing ness to accept a challenging task (Baron and Kenny, 1986). The effect of AI application was reduced to non-significance (from *b* = 0.748, *p* = 0.037, to *b* = 0.434, *p* = 0.217) when robot trust was included in the equation, and robot trust was a significant predictor of willingness to accept a challenging task (*b* = 0.429, *p* = 0.000). The Sobel test confirmed the mediation (*z* = 2.365, *p* = 0.018), indicating that robot trust mediated the relationship between robot gender and willingness to accept a challenging task.

## Discussion

This study investigates the impact of gender-specific anthropomorphism in robots on human motivation to undertake challenging tasks within human-robot collaborative settings. Through two controlled experimental studies, we demonstrated that participants interacting with female-gendered robot counterparts exhibited a significantly higher willingness to accept challenging tasks compared to those interacting with male-gendered robot counterparts. Our findings reveal a measurable gender-based disparity in challenge acceptance behavior, suggesting that anthropomorphic gender cues in robotic interfaces may substantially influence human decision-making patterns during collaborative task scenarios.

Research consistently demonstrates that gender stereotypes automatically manifest in HRI, with feminine-gendered robots perceived as warmer yet less competent compared to their masculine counterparts, which are typically viewed as more authoritative (Borau, 2024; Eyssel and Hegel, 2012; Perugia and Lisy, 2023). This gender-competence stereotypes have been observed across multiple studies (Duan et al., 2024; Loideain and Adams, 2020; Otterbacher and Talias, 2017; Perugia and Lisy, 2023). However, our experimental findings showed that participants collaborating with a feminine-gendered robot demonstrated significantly greater willingness to undertake challenging tasks than those interacting with a masculine-gendered robot, despite showing no statistically significant difference in their evaluations of robotic capabilities. This suggests that gender stereotypes, while cognitively accessible, may not directly predict functional outcomes in collaborative HRI contexts.

In our experiments, robots were assigned assistive roles, and female-gendered robots received significantly higher trustworthiness ratings compared to their male-gendered counterparts. This finding aligns with prior research demonstrating that robots endowed with female attributes (e.g., voices, personas) are consistently preferred for assistive roles (Nass and Brave, 2005) and perceived as warmer and more effective collaborators in task-oriented contexts (Carpenter et al., 2009; Sandygulova and O’Hare, 2018; Siegel et al., 2009). Our results reinforce the established correlation between feminized robot designs and perceptions of warmth and cooperative utility in caregiving or service-oriented interactions.

Over the past decades, foundational theories of human motivation (Alderfer, 1969; Deci and Ryan, 1985; Hackman and Oldham, 1976; Herzberg et al., 1959; Locke and Latham, 1990; McClelland, 1961; McGregor, 1960; Vroom, 1964) have established critical frameworks for understanding workplace behavior. As contemporary organizational environments increasingly integrate robotic partners, understanding motivational dynamics in HRI becomes crucial for informing effective workplace technology integration. Our research extends this theoretical foundation by revealing significant gender anthropomorphism effects in task engagement. Consistent with prior work demonstrating that interpersonal trust enhances employee task motivation (Costa et al., 2018; Edmondson, 1999), our findings suggest that robot gender promotes task motivation through the mediating role of trust toward robots in HRI. Our finding underscores the importance of intentional gender design in robotic interfaces, particularly for complex task allocation systems where employee engagement directly impacts operational outcomes.

The present study has several limitations: Firstly, its reliance on a convenience sample of university students may restrict the generalizability of findings to broader populations. Given potential disparities in technological familiarity and cognitive schemas about robotics between student groups and the general public, this sampling approach risks overestimating technology acceptance rates. Future investigations should consider encompassing diverse age groups, professional backgrounds, and cross-cultural comparison to validate the external validity of the results. Second, the experimental paradigm’s dependence on static visual stimuli (i.e., images) inadequately captures multimodal interaction dynamics inherent to physical human-robot collaboration. Future research should employ real robots in naturalistic settings to simulate authentic collaborative contexts. Third, the study exclusively compared female-gendered and male-gendered robots, both characterized by high anthropomorphism. To fully elucidate the interaction between robot gender and anthropomorphism on individuals’ motivation to undertake challenging tasks, future work should include robots with moderate anthropomorphism and explore gender-neutral designs. Finally, Study 2 did not collect actual performance data. Future research using a similar methodology should include such measures to provide deeper insight into participants’ task motivation.

In summary, this study investigated the impact of gender-specific anthropomorphism in robots on human motivation to undertake challenging tasks. As hypothesized, our two studies indicate that participants interacting with a female-gendered robot demonstrated a higher propensity to accept a challenging task compared to those engaging with male-gendered counterparts. Despite the contributions of our research, potential limitations should be acknowledged, which might limit the generalizability and applicability of our results. This research provides insights to the design of collaborative robots by highlighting the importance of gender cues in optimizing human engagement and performance in task-oriented settings.

## Data Availability

The raw data supporting the conclusions of this article will be made available by the authors, without undue reservation.

## Footnotes

^1^ https://www.abotdatabase.info
